# Using Three Indirect Measures to Assess the Role of Sexuality-Related Associations and Interpretations for Women’s Sexual Desire: An Internet-Based Experimental Study

**DOI:** 10.1007/s10508-020-01897-3

**Published:** 2021-04-12

**Authors:** Lisa Zahler, Milena Meyers, Marcella L. Woud, Simon E. Blackwell, Jürgen Margraf, Julia Velten

**Affiliations:** Clinical Psychology and Psychotherapy, Mental Health Research and Treatment Center, Faculty of Psychology, Ruhr University Bochum, Massenbergstr. 9-13, 44787 Bochum, Germany

**Keywords:** Sexual desire, Sexuality-related interpretations, Cognitive biases, Indirect measures, Automatic associations

## Abstract

Theoretical models emphasize the role of both automatic appraisals (i.e., associations) and conscious appraisals (i.e., interpretations) for sexual desire. Studies on sexuality-related appraisals have not combined self-report measures and experimental paradigms in order to compare the relevance of associations or interpretations. The aim of this study was to assess the relative contribution of both associations and interpretations to the explanation of low sexual desire in women. Toward this goal, indirect measures assessing associations (via a Single Target Implicit Association Test [STIAT]) and interpretations (via a Scrambled Sentences Test [SST] and a scenario task) were administered in a sample of 263 women (*M*_age_ = 27.90, SD 8.27) with varying levels of sexual desire and different sexual orientations (exclusively heterosexual women: 54.6%). Negative sexuality-related interpretations as assessed with two variants of the SST as well as the scenario task added to the explanation of lower sexual desire in women. Negative associations as measured with the STIAT were predictive of lower sexual desire only in women who did not indicate an exclusively heterosexual orientation. In this study, sexuality-related interpretations were more relevant to women’s sexual desire than automatic associations. Future studies should assess the causal mechanism underlying sexuality-related interpretations (e.g., by evaluating whether these can be changed via cognitive bias modification techniques or psychological treatments).

## Introduction

Difficulties with sexual functioning are common among women in the general population (Laumann et al., 1999), with prevalence rates ranging from 43% (Shifren et al., 2008) to 51% (Mitchell et al., 2013). Sexual difficulties can have detrimental effects on women’s quality of life and are associated with decreased physical and psychological health, overall well-being, and partnership satisfaction (Velten & Margraf, 2017). One of the most common sexual difficulties in women is low sexual desire (Shifren et al., 2008), which can include a lack of interest in sexual activities, reduced sexual initiative , or a lack of receptiveness to a partner’s attempts to initiate sex (American Psychiatric Association, 2000). Low sexual desire also commonly co-occurs with disturbances in other aspects of the sexual response, e.g., reduced arousal, problems with orgasm (Laumann et al., 1999; Mitchell et al., 2013).

### The Role of Appraisals for Sexual Desire

**Fig. 1:**
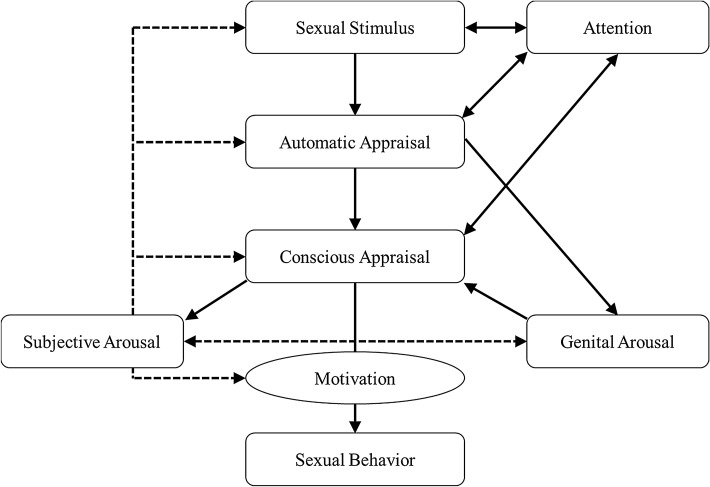
An emotion-motivational model on sexual arousal based on the models of Barlow (1986), Janssen et al. (2000), and Öhmann (1993) by Dewitte (2016) Printed with permission from Wiley

Theoretical models of the etiology and maintenance of sexual difficulties (Barlow, 1986; Janssen et al., 2000; Toates, 2009) emphasize the relevance of cognitive processes (e.g., attention, appraisal, or memory) which operate at different stages of awareness and control (Corr, 2010). Janssen et al.’s information processing model, for example, describes the relevance of both automatic and conscious appraisals for sexual arousal. Building on earlier models, Dewitte’s (2016) emotion-motivational model (see Fig. 1) provides a theoretical framework for how automatic appraisals, also called associations (Roefs et al., 2011), and more conscious, deliberate appraisals, also called interpretations (Hirsch et al., 2016), interact with attentional processes in influencing sexual motivation. This model focuses on cognitive processes as precursors of sexual motivation—which can be described as an energizing force that impels individuals to seek out sexual behavior or to respond to sexual stimulation—and has been used synonymously to sexual desire (Kaplan, 2013).

To illustrate the pathways trough which appraisals might be involved in the facilitation of sexual desire, two examples are provided. In a sexually healthy woman without sexual desire concerns, a sexual stimulus (e.g., her naked partner taking a shower) can pre-attentively capture her attention and she may automatically appraise it as sexually meaningful and rewarding. These positive automatic associations are likely to be followed by increased cognitive elaboration and interpretation and she may, e.g., think about a recent sexual encounter. She may also experience first signs of genital sexual arousal (e.g., a feeling of warmth, vaginal lubrication). Her positive appraisals as well as her perception of a genital response might then trigger subjective feelings of sexual arousal and desire, which can feed into further increases in genital arousal. Combined, these cognitive and physiological changes increase her desire or motivation to have sex with her partner (Dewitte, 2016). In contrast, a woman with low sexual desire might appraise the situation negatively and as a result, not experience sexual desire. First, she may not automatically appraise her partner’s naked body as a desirable sexual cue. Then, she might further elaborate and remember a recent conflict about her lack of sexual initiative or an unpleasurable sexual encounter. These associations and interpretations may not trigger genital arousal and her desire for sex remains low. Thus, both automatic and conscious cognitive appraisals might nudge her to leave the situation rather than approaching her partner for sex.

### Assessment of Sexuality-Related Associations and Interpretations

The most common way to assess conscious sexuality-related interpretations is the use of direct measures (i.e., self-report questionnaires). Using self-report, studies have shown that decreased sexual desire is associated with negative sexual cognitions and concerns regarding one’s sexual functioning (e.g., Bancroft et al., 2003; Carvalho & Nobre, 2010; Dennerstein et al., 1999; Silva et al., 2016; Velten & Brotto, 2017). Despite the valuable insights gained by self-report measures, there are several limitations, such as demand effects (Richman et al., 1999). Another disadvantage of self-report measures is that they do not capture more automatic appraisals of sexual stimuli, which participants might not be aware of or might be reluctant to share. Indirect measures, which use a behavioral performance to deduct the concept of interest, for example, via reaction times, can be used as both an alternative and complementary approach to capture sexuality-related associations. A variety of reaction-time measures including dot-detection, line orientation (Kagerer et al., 2014), or spatial cueing tasks (Imhoff et al., 2020) have been applied to investigate cognitive processes with respect to sexual outcomes. While many tasks target attention rather than appraisals, the (Single Target) Implicit Association Test was developed to specifically assess implicit associations (Ouimet et al., 2009).

#### Single Target Implicit Association Test (STIAT)

The STIAT (Wigboldus et al., 2004) measures such automatic appraisals by having participants categorize stimuli into different categories as quickly as possible. Participants’ reaction times are used as a performance-based measure to infer the evaluation of the presented stimuli indirectly. Generally, it is assumed that strongly associated stimuli are processed more quickly compared to weakly associated stimuli, which is reflected in participants’ categorization speed (Bargh, 1996; Greenwald et al., 2009). Different versions of the STIAT have been used to investigate the role of negative automatic appraisals for low sexual desire in women. Brauer et al. (2012) employed a STIAT to assess whether women with Hypoactive Sexual Desire Disorder (HSDD; American Psychiatric Association, 2000) show stronger negative associations with pictures showing sexual acts, compared to healthy controls. Results showed that women with HSDD had slower reaction times when sexual pictures shared the same response key with positive versus negative attributes, compared to healthy controls. This indicated that women with HSDD exhibited less positive appraisals of sexual stimuli. To distinguish the affective valence (i.e., how much women like sex) from the motivational value (i.e., how much women want sex) of sexual stimuli, van Lankveld et al. (2018a) employed two different STIATs in a sample of women with sexual complaints (including low sexual desire) and healthy controls. The STIATs were used to assess whether women with sexual complaints automatically associated pictures displaying sexual intercourse with either the categories positive/negative (STIAT-liking; representing affective valence) or with the categories I want/I do not want (STIAT-wanting; representing motivational value). Women with sexual complaints, compared to healthy controls, were faster when sexual intercourse pictures shared one response key with the motivational category “I do not want.” However, when sexual pictures shared one response key with the affective valence category “negative,” results indicated no difference in response latencies between women with and without sexual difficulties. Further, automatically associating sexual cues with the concept of “wanting” was associated with higher levels of sexual functioning. Dewitte (2015) used two similar versions of STIAT-liking and STIAT-wanting to investigate whether affective valence and motivational value of sexual stimuli depended on whether women were in a romantic or in a sexual mood. In their sample of 78 women, they found evidence for a context-dependency of the STIAT-wanting as female participants showed a greater implicit wanting of sex after watching a romantic as compared to a sexual film clip. Although this study did not directly implicate the role of wanting in low sexual desire, the author concluded that focusing on specific components of sexual appraisals (e.g., liking or wanting) and taking contextual factors such as mood into consideration may be useful to advance knowledge on sexual dysfunctions (Dewitte, 2015). Concerning the methodological details of sexuality-related STIATs, studies often used sexual pictures (Borg et al., 2010; van Lankveld et al., 2018a, b) or words (e.g., intercourse, orgasm) as erotic targets. As many studies have excluded non-heterosexual participants, however, it is unclear whether the use of heteronormative stimuli influences reaction times of female participants with different sexual orientations. Evidence from one study using the STIAT pointed in this direction as associations of lesbian (but not of straight) women were consistent with their self-reported preferred stimulus (Snowden & Gray, 2013).

To sum up, research on the STIAT-wanting suggests that automatic appraisals concerning the motivational value of sex are likely to be relevant for low sexual desire concerns in women. Using these motivational categories, women with low desire are expected to show faster categorization times for sexual stimuli paired with a label indicating a lack of incentive-motivation (“I don’t want”) as compared to a label indicating a high motivational pull (“I want”) (Dewitte, 2016; van Lankveld et al., 2018a).

#### Scrambled Sentences Test (SST)

Interpretations or conscious appraisals of sexual stimuli cannot only be measured via self-report but also by using indirect tasks which reduce participants’ volitional control over responses (e.g., Rude et al., 2002). One such measure is the SST (Rude et al., 2001; Wenzlaff & Bates, 1998) that requires participants to sort six words into a grammatically correct sentence, using only five words. Depending on the omitted word, the resulting sentence can have, e.g., a positive or negative valence or can reflect a disorder-specific interpretation (e.g., interpretation of body-symptoms in a panic-related or a neutral way). The SST has already been established as a valid task for measuring interpretation biases in, e.g., depression or panic disorder (Sanchez et al., 2015; Zahler et al., 2020) but has not yet been applied to research assessing sexual difficulties. In the context of this study, the goal was to create a novel sexuality-related SST that taps into the two components of appraisals mentioned above (e.g., affective liking vs. motivational wanting; see Method for details on the SST).

#### Scenario Task

Another alternative to assessing interpretations is using a scenario-based approach (Velten et al., 2019; Woud et al., 2012a). In the context of sexual difficulties, Velten et al. (2019) developed open-ended, ambiguous sexual scenarios, which participants had to complete by generating an individual ending for each scenario. Independent raters coded the endings on different dimensions (i.e., the presence of sexual problems, sexual communication, and sexually explicit language) and results showed that women with sexual difficulties generated more endings that indicated sexual problems, less sexual communication with a partner, and less use of sexually explicit language. Hence, using such a scenario-based approach, the authors provided first evidence for the negative interpretation of ambiguous sexuality-related scenarios in participants with low sexual function.

To summarize, the emotion-motivational model (Dewitte, 2016) supports the relevance of associations and interpretations in the etiology and maintenance of low sexual desire. Studies using self-report measures have shown that women with low sexual desire interpret sexual stimuli in a negative way. However, these studies are also subject to methodological difficulties and may not (adequately) capture more automatic or pre-attentive associations. Indirect measures may provide a complementary approach and studies using these measures found evidence for negative automatic associations (van Lankveld et al., 2018a) and interpretations (Velten et al., 2019) in women with low levels of sexual desire and low sexual functioning. Given the importance of appraisals for sexual motivation and sexual functioning, and the small number of studies having investigated the relative contribution of associations and interpretations to problems concerning low sexual desire, further studies are needed. The aim of the current study was to extend previous findings by using three different indirect measures (i.e., STIAT, SST, and scenario task) in a larger sample of women with mixed levels of sexual desire. By validating the previously used STIAT and scenario task in a non-clinical sample and developing a novel task (i.e., the SST) that taps into both affective and motivational components of sexual appraisals, this study contributes to the refinement of conceptualizations of associations and interpretations for low sexual desire in women.

### The Present Study

To examine sexuality-related associations, a STIAT-wanting was used. With regard to sexuality-related interpretations, the SST, a promising and pioneering tool for interpretation bias in sexual difficulties, as well as three scenarios from a scenario-based approach (Velten et al., 2019) were employed. We recruited women from the community with different levels of sexual desire. Hypothesis 1 was that women with lower sexual desire would show faster categorization times during the STIAT-wanting when erotic stimuli share the same response key with the motivational category “I do not want” compared to when the erotic stimuli share the same response key with the motivational category “I want.” Hypothesis 2 was that lower sexual desire would be associated with more negative sexuality-related sentences (SST-liking) as well as fewer sentences indicative of the motivational wanting of sex (SST-wanting). Hypothesis 3 was that lower sexual desire would be associated with more endings indicative of sexual problems in the scenario task. In addition, we investigated whether the indirect measures would add to the explanation of sexual desire above and beyond the explicit evaluation of the sexual pictures in the STIAT-wanting, sexuality-related personal distress (i.e., an explicit interpretation of one’s sexual life and sexual difficulties), and partnership status, and whether results on the STIAT-wanting would be affected by women’s sexual orientation.

## Method

### Participants

Participants were recruited via different channels, e.g., social media, postings on the university webpage, and online discussion boards to increase the diversity of the sample. In addition, participants from previous studies who had provided consent to be contacted for future studies were invited via e-mail. Participants were required to be female, 18 years or older, and had to have access to a computer or laptop with a computer mouse.

Of the 263 tested participants in the study, 261 provided their age (*M*_age_ = 27.90, SD 8.27, range = 18 to 61) and their country of origin. Of these participants, 252 (96.6%) were born in Germany and 9 (3.4%) were born in a country other than Germany. Of the 252 participants born in Germany, 41 (16.3%) had at least one parent or grandparent born in a country other than Germany. Of the 261 participants who indicated their relationship status, 147 participants (56.32%) were in a monogamous relationship, 19 (7.28%) in an open, non-monogamous relationship, 57 (21.84%) indicated being single with sexual encounters during the past 6 months, 22 (8.43%) indicated being single without sexual encounters during the past 6 months, and 16 (6.13%) entered a different relationship status. With regard to sexual orientation, 142 (54.4%) women indicated being exclusively heterosexual (Kinsey 0), and 72 (27.6%) indicated being predominantly heterosexual and only incidentally homosexual (Kinsey 1). Further, 16 (6.1%) participants identified as predominantly heterosexual, but more than incidentally homosexual (Kinsey 2), while 7 (2.7%) identified as equally heterosexual and homosexual (Kinsey 3). Being predominantly homosexual, but more than incidentally heterosexual (Kinsey 4) was indicated by 1 (0.4%) participant, while being predominantly homosexual and only incidentally heterosexual (Kinsey 5) was indicated by 10 (3.8%) participants. Further, 5 (1.9%) participants identified as exclusively homosexual (Kinsey 6) and 8 (3.1%) participants indicated a different sexual orientation, such as pansexual. Most participants were highly educated, with 241 participants (91.63%) finishing High School and 146 participants (55.51%) having obtained a University degree. The majority of participants was working either full time (*n* = 77; 29.28%), part time (*n *= 43; 16.35%) or were enrolled as university students (*n *= 119; 45.25%).

### Procedure

Interested participants were informed about the study’s contents and then provided informed consent via a digital consent form. Next, participants provided their demographic information and their e-mail address. Two e-mails were then sent to the participants, which included links to the two parts of the study. The link in the first e-mail took participants to the reaction-time paradigms (i.e., the STIAT and SST, in random order). To complete these, participants needed to download a simple computer program (i.e., Inquisit 5 Web App). Instructions on how to install the program were also provided in the first e-mail, and in the download information on the website of the computer program. Once participants completed the STIAT and SST, participants were instructed to use the link provided in the second e-mail, which took participants to the online survey comprising the self-report measures. The scenario task was completed as part of this survey. The total duration of the study was approx. 45 min. The independent Ethics Committee of the Faculty of Psychology at the first author’s university (ethical approval number: 073-E2) approved all study procedures. All participants provided online informed consent as part of the initial screening.

### Clinical Measures

#### Sexual Interest and Desire Inventory Female (SIDI-F)

The SIDI-F (Clayton et al., 2006) is a clinician-rated instrument consisting of 13 items measuring sexual desire in women. It also includes one non-scored item assessing intercourse frequency. In the present study, we administered the SIDI-F as a self-report questionnaire. This adapted version of the SIDI-F has shown good internal consistency in previous studies, which administered it in samples of women with low sexual desire (Brotto & Basson, 2014; Paterson et al., 2017). Depending on the respective items, these were rated from 0 to 3, 4, or 5, with the total score ranging from 0 to 51 and higher scores indicating more sexual interest. The presence of clinically relevant low levels of sexual desire was determined using the cutoff score of 33 (Clayton et al., 2010). In the present study, internal consistency of the total scale was good with *α* = .81.

#### Female Sexual Distress Scale-Revised (FSDS-R)

The 13-item FSDS-R (DeRogatis et al., 2008) was used to measure sexuality-related personal distress. On the FSDS-R, women indicate the degree to which they experience a variety of negative cognitions (e.g., worry, guilt) and emotional reactions (e.g., unhappiness, anger) regarding their sexual life in general, sexual problems, and sexual relationships. Responses were made on a 5-point Likert scale ranging from 0 = “never” to 4 = “always,” with a total score ranging from 0 to 52 and higher scores indicating more sexuality-related personal distress. A total score of equal to or above 11 on the FSDS-R is recommended as cutoff score to establish the presence of sexually related personal distress (DeRogatis et al., 2008). The FSDS-R has shown high internal consistency, high test–retest reliability, and good discriminant validity (DeRogatis et al., 2008). In the present study, internal consistency of the scale was excellent with *α* = .91.

### Experimental Tasks

#### Single Target Implicit Association Test (STIAT)

The stimulus material used for the STIAT-wanting was partly based on the study by van Lankveld et al. (2018a). In their study, the two attribute categories “liking” and “wanting” were used. However, results showed that only STIAT-wanting but not the STIAT-liking differentiated between female participants with and without sexual problems. Hence, we only used the attribute category wanting, which was labeled “I Want” versus “I Do Not Want.” The target category label was “Sex.” There were 4 words per attribute category: The attribute category “I Want” included four positive words (Humor, Health, Gift, Peace), and the attribute category “I Do Not Want” included four negative words (Hatred, Disease, Pain, War).^1^ Target stimuli were four erotic pictures that were derived from the International Affective Picture System (IAPS), showing heterosexual couples engaging in sexual activities (numbers 4658, 4672, 4680, and 4694).

Participants were instructed to sort the pictures and words according to their categories by pressing the response keys D or K on their keyboard. In the top left and right corners of the monitor, the attribute category labels were displayed and remained there during the entire task. The target category label was displayed either beneath the “I Want” or beneath the “I Do Not Want” label, depending on the task assignment. Stimuli (words and pictures) appeared in the center of the screen. The inter-trial interval was 250 ms. When an incorrect response was given, a red cross appeared on the screen and participants had to re-categorize the stimulus into the correct category. To familiarize participants with the task, they practiced the categorization of the attribute words during the initial attribute practice phase (20 trials). Then, the first combined block was presented, i.e., the target category label appeared additionally in the left or right corner of the monitor (depending on the task assignment), and participants were asked to sort attribute and target words according to their respective categories (60 trials). During this block, targets and sexual pictures/positive attributes shared the same response key (compatible block; sex-positive combination). During the subsequent second combined block, the target category label was in the opposite position on the screen, that is, the task assignment was reversed (60 trials). During this block, targets and sexual pictures/negative attributes shared the same response key (incompatible block; sex-negative combination). The order of task assignment was counterbalanced, i.e., half the participants started with the incompatible and then the compatible block, and the other half started with the incompatible block, then the compatible block. In all experimental blocks, the computer program selected all attributes and targets randomly out of the stimuli pool. In this study, the Spearman–Brown split-half reliability was good with *r* = .85.

#### Explicit Evaluation Scale of Erotic Stimuli (EEES)

In order to obtain an explicit evaluation of the four erotic pictures used in the STIAT, participants were asked to rate these pictures using two dimensions, i.e., valence and arousal on 9-point Likert scales ranging from “very negative” to “very positive” and from “not arousing at all” to “very arousing,” respectively. For each participant, scores on each dimension were summed to a total score, with higher scores indicating a positive valence and higher levels of sexual arousal. This procedure was adapted from van Lankveld et al. (2018a).

#### Scrambled Sentences Task (SST)

For this study, a novel sexuality-related SST was developed based on previous studies using this task for other mental health issues (e.g., panic disorder; Zahler et al., 2020). Sentences were created by the senior author, who is a clinical psychologist with experience in treating women with low sexual desire. Thus, the goal was to create sentences relevant for women’s sexual desire and offer (at least) two options that are comparably common in the German language. Scrambled sentences each consisted of six words that should be unscrambled using only five words. In a first variant of the SST, scrambled sentences (e.g., “pleasure during frustration I sex feel”) could be used to create either an interpretation that describes sex as positive or pleasurable (e.g., “I feel pleasure during sex”) or an interpretation with sex as frustrating, negative, or disappointing (e.g., “I feel frustration during sex”). To tap into the incentive value or the motivational wanting of sex, scrambled sentences (e.g., have chocolate desire for sexuality I) were written to allow for a sexual (e.g., “I have a desire for sexuality”) or a neutral/nonsexual (e.g., “I have a desire for chocolate”) interpretation.

Sentences were then piloted in a small student sample and 20 scrambled sentences were selected for this study. Two mean scores were calculated: One reflecting a more negative (vs. positive) resolution of sentences (SST-liking), the other one reflecting preference for nonsexual versus sexual sentences (SST-wanting; see Supplemental material for a complete list of sentences).

Each trial started with the presentation of a fixation cross for 500 ms, and 500 ms later, the scrambled sentence appeared. Participants were instructed to unscramble the sentences by clicking with the mouse on only 5 words in an order that would create a grammatically correct statement. As soon as participants clicked on a word, a white number appeared above the word to indicate the word’s position. Once an order was chosen, participants were not able to change it. The scrambled sentences appeared consecutively, and participants were given 10 s for the completion of each sentence. In case the time limit was exceeded, the next sentence was presented. The task started with a practice phase including five neutral scrambled sentences. The stimuli of the practice trials were adapted from Zahler et al. (2020). Afterward, the experimental phase began. As suggested by Wenzlaff and Bates (1998), participants were presented with a six-digit number prior to the task, which they had to keep in mind during the task and reproduce afterward (cognitive load). These numbers were presented for 10 s. It is assumed that the effect of such a cognitive load can decrease the influence of participants’ cognitive regulation mechanisms during the task (Rude et al., 2003). In this study, split-half reliability was good, with *α* = .86.

#### Scenario Task

In this study, we used three ambiguous, open-ended sexual scenarios, which were generated and validated in a previous study (Velten et al., 2019). The scenarios consisted of three sentences each and ended abruptly. Participants were instructed to read and imagine the scene described in each sentence, and to complete each sentence into a grammatically correct and meaningful manner (for more details, see Velten et al., 2019). One scenario targeted participants’ general satisfaction with their sexual life: “After a delicious dinner, I am sitting around with a group of friends. After a few drinks, we start talking about sex. A friend addresses me directly and asks: “How’s it going with you in bed?” I blush and say: ….” In the second scenario, women’s satisfaction with their latest sexual encounter was targeted: “It’s the weekend and I enjoy not having to get up early. Cuddled up to my partner, I feel the warmth of their body. When they wake up, they say “Honey, the sex last night was….” In a third scenario, a situation involving a sexual difficulty was described and assessed how participants’ partners would react in this situation: “During sex I notice that my partner’s breathing is going faster and that they are very aroused. I feel, however, that my body is responding differently. After a little while, my partner also notices this and….” The scenario endings were rated by two external raters, who rated whether or not endings described a sexual problem (e.g., low sexual desire), with 0 = “not present” and 1 = “present.” A mean total score was calculated by summing up the ratings of the two raters across the three scenarios and dividing them by two. Hence, scores could range between 0 and 3. For more details on the scenario task, see Velten et al. (2019). In this study, the Spearman–Brown split-half reliability was poor with *r* = .43.

### Statistical Analysis

#### Single Target Implicit Association Test

We aggregated and analyzed the STIAT data according to the procedures described by Bluemke and Friese (2008), and included only trials of the combined blocks (experimental trials) in the analysis. Prior to the analysis, two participants were excluded due to technical difficulties. Error trials were also excluded from the analysis (4.4%), and response latencies below 300 ms (0.1% of values) and above 3000 ms (1.1% of values) were recoded to these respective values. This means that lower values were recoded to 300ms and higher values to 3000 ms. In addition, we excluded another 15 participants who had made 20% or more errors in any of the individual blocks (mean error rate after exclusion 6.6%). In accordance with Bluemke and Friese (2008), we omitted the first trial of each block as a practice trial. To control for variabilities in response latencies, participants’ mean latencies were divided by the overall SD for that participant. Then, we generated a D-score by subtracting the mean latencies of the combined incompatible block (sex-negative combination) from the combined compatible block (sex-positive combination). This means that quicker reaction times for sex-negative combinations compared to sex-positive combinations are represented by a positive D-score.^2^

#### Scrambled Sentences Test

We excluded all trials that exceeded the 10 s time frame (8.8%) and all trials that were grammatically incorrect (4.8%). This was done by manual inspection of the data. We then created two scores reflecting the total number of correct sentences for the affective (i.e., positive/negative statements about sexuality) or motivational (i.e., sexual vs. nonsexual sentences) categories of the SST for each participant. These scores were then used to calculate two ratios by dividing the number of negative or neutral sentences by the total number of all correctly solved sentences per category. Consequently, higher values were indicative of generating more negative (vs. positive) interpretations in the SST-liking and more and neutral (vs. sexual) interpretations in the SST-wanting.

#### Scenario Task

The second author and a Masters-level Clinical Psychology student rated the endings of the three sexuality-related scenarios. A coding schema was developed by the senior author. If a scenario continuation was incomplete or incomprehensible, or included any meta-comments, it was not included in the analysis (6.5%). There was high agreement between the two raters’ judgments, with *κ *= .81.

### Statistical Analysis

All analyses were conducted using SPSS 25. We report the means and standard deviations of the questionnaires and reaction tasks as descriptive values. Bivariate Pearson’s *r* correlation coefficients between predictors and outcome (i.e., sexual desire) are reported as a preliminary analysis. To test our hypotheses, a hierarchical linear regression analysis was calculated. In Step 1, the three indirect measures were added as predictors. To control for a potential confounding effect of the heterosexual stimulus material used in the STIAT, sexual orientation (dichotomized as exclusively heterosexual orientation vs. other sexual orientation), as well as an interaction between the D-score and sexual orientation were included. In Step 2, the explicit evaluation of sexual stimuli used in the STIAT as well as sexuality-related personal distress were added to examine whether the indirect measures added to the explanation of sexual desire over and above explicit sexuality-related appraisals. All predictors were mean centered. Bootstrapped 95% confidence intervals as well as semipartial *R*^2^ (small: .02 ≤ .13, medium: .13 ≤ .26; or large: ≥ .26; Cohen, 1988) are reported as effect sizes for predictors in the regression analysis. The dataset used for the analysis can be accessed at the following website: osf.io/ruksm/.

## Results

### Sample Characteristics

On average, participants scored 33.00 (SD 8.15) on the SIDI-F, and the scores of 142 participants (55.25%) were in the clinical range indicating low levels of sexual desire (see Table 1). Sexual distress measured with the FSDS-R was on average 9.75 (SD 9.12) and 97 women (37.74%) scored above the clinical cutoff. These findings speak to the representativeness of the study sample, in that low sexual desire and sexual distress are prevalent in the general female population (Mitchell et al., 2013). In the SST-liking, 2090 positive and 618 negative sentences were generated. The SST-wanting led to the generation of 1258 sensual and 1289 neutral/nonsexual sentences. Based on the average scores generated by the two external raters, the sum score for the three scenario continuations with regard to sexual problems was 0.33 (SD 0.56, range 0-3). Table 1 provides an overview of these descriptive statistics as well as of the reaction-time paradigms used in this study, i.e., the STIAT-wanting, SST-liking, and SST-wanting.

**Table 1:** Sample characteristics

Measure	*N*	*M*	*SD*	*Min*	*Max*
Sexual Interest and Desire Inventory Female (SIDI-F-SR)^a^	257	33.00	8.15	6.00	46.00
Female Sexual Distress Scale-Revised^b^	257	9.75	9.12	0.00	39.00
Single Target Implicit Association Test (STIAT)	244	− 0.09	0.38	− 1.03	0.99
Explicit Evaluation Scale of Erotic Stimuli (EEES)^c^	254	8.26	1.61	1.50	11.00
Scrambled Sentences Test-Liking (SST-liking)	263	0.09	0.12	0.00	0.56
Scrambled Sentences Test-Wanting (SST-wanting)	263	0.23	0.11	0.00	0.54
Scenario task^d^	246	0.33	0.56	0.00	3.00

Absolute ranges: ^a^0–51; ^b^0–52; ^c^0–24; ^d^0–3

### Preliminary Analysis

#### Correlation of Experimental Tasks

The overall pattern of correlations between the experimental tasks was comparable for women with exclusively heterosexual and those with other sexual orientations. As expected, the D-score of the STIAT-wanting scored showed a small, negative correlation with EEES (see Table 2) indicating that more negative implicit associations were related to more negative explicit ratings of sexual pictures. In addition, the D-score showed a small, positive correlation with SST-wanting suggesting that both indirect measures that aimed to assess the motivational wanting of sex corresponded with each other. D-scores were not significantly associated with the scenario task. Further, more negative interpretations on the SST-liking were associated with more negative endings on the scenario tasks indicative of sexual difficulties, while more nonsexual interpretations on the SST-wanting were not significantly correlated with the scenario task.

**Table 2:** Bivariate correlations between implicit measures, explicit measures, confounding variables, and sexual desire for exclusively heterosexual women (top-right) and women with other sexual orientations (bottom-left)

	SIDI-F-SR	STIAT-wanting	SST-liking	SST-wanting	Scenario task	EEES	FSDS-R
Sexual Interest and Desire Inventory Female (SIDI-F-SR)	1	.10	− .63^***^	− .29^***^	− .37^***^	.45^***^	− .53^***^
Single Target Implicit Association Test (STIAT-wanting)	− .18	1	.07	.10^*^	− .04	− .12	− .18^*^
SST-liking	− .57^***^	.01	1	.27^**^	.34^***^	− .45^***^	.48^***^
SST-wanting	− .28^**^	.16	.27^**^	1	.08	− .35^***^	− .05
Scenario task	− .36^***^	− .19	.35^***^	− .07	1	− .18^*^	.37^***^
Explicit evaluation of erotic stimuli (EEES)	.17	− .21^*^	− .23^*^	− .25^**^	− .06	1	− .12
Sexual distress (FSDS-R)	− .53^***^	.07	.53^***^	.13	.40^***^	.004	1

* *p* < .05. ** *p *< .01. *** *p *< .001

#### Correlations of Experimental Tasks and Sexual Desire

Sexual desire showed a nonsignificant positive correlation with the D-score of the STIAT-wanting in exclusively heterosexual women (*r* = .10) but showed a negative correlation in the remaining participants (*r* = −.18). A post hoc test (Eid et al., 2011, p. 547) showed that these correlations differed significantly from each other (*z* = 2.14, *p *= .016). In other words, the expected relationship of more negative associations measured with the STIAT being associated with lower sexual desire was *not* found in exclusively heterosexual women but showed a trend toward significance in women with other sexual orientations. Another difference emerged for the explicit ratings of the sexual pictures of the STIAT: A more positive evaluation of the pictures was associated with higher sexual desire, but only in exclusively heterosexual women. The remaining correlations were comparable across sexual orientation groups. Higher sexual desire was associated with more positive interpretations in the SST-liking, more sexuality-related interpretations on the SST-wanting, and fewer completed scenarios indicative of sexual problems. Overall, the pattern of results between the three experimental tasks and sexual desire was as expected with the exception that the D-score was not associated with sexual desire in exclusively heterosexual women.

### Explanation of Sexual Desire

In Step 1, the model explained 43.6% (adjusted *R*^2^, *p* < .001) of variance in sexual desire. While neither the STIAT nor sexual orientation predicted sexual desire, their interaction term was a negative predictor (see Table 3). A post hoc simple slope analysis indicated that the hypothesized relationship between negative associations as measured with the STIAT-wanting and lower sexual desire was found in neither of the sexual orientation groups but showed a trend in the expected direction in women who endorsed other sexual orientations (exclusively heterosexual: *b* = 2.15, SE 1.86, *t* = 1.16, *p* = .249; other sexual orientations: *b* = −3.59, SE 2.00, *t* = −1.80, *p* = .074). As expected, both SST-liking and SST-wanting as well as the scenario task were negative predictors suggesting that more negative sexuality-related interpretations were associated with lower sexual desire. In this model, effects on sexual desire were small with the exception of SST-liking which had a medium effect (*R*^2^ = .22).

**Table 3:** Prediction of sexual desire as measured with the Sexual Interest and Desire Inventory Female (SIDI-F-SR)

Predictors	B	SE	Sig.	Bootstrap CI		*R*^2^
Step 1				LL	UL	
Constant	32.88	0.40	< .001^***^	32.09	33.68	
STIAT (D-score)	0.37	1.08	.732	− 1.76	2.50	< .01
Sexual orientation (Kinsey 0 vs. other)	0.60	0.83	.474	− 1.04	2.23	< .01
STIAT * Sexual orientation	− 5.75	2.14	.008^**^	− 9.98	− 1.53	.02
SST-liking (positive sentences = 0, negative sentences = 1)	− 35.22	3.75	< .001^***^	− 42.62	− 27.83	.22
SST-wanting (sexual sentences = 0, nonsexual sentences = 1)	− 11.86	4.11	.004^**^	− 19.96	− 3.76	.02
Scenario task	− 2.73	0.76	< .001^***^	− 4.22	− 1.24	.03
Step 2						
Constant	32.83	0.37	< .001^***^	32.10	33.57	
STIAT (D-score)	0.36	1.00	.716	− 1.60	2.33	< .01
Sexual orientation (Kinsey 0 = 1, other sexual orientation = 2)	1.43	0.78	.067	− 0.10	2.96	< .01
STIAT * Sexual orientation	− 3.48	2.01	.085	− 7.43	0.48	< .01
SST-liking (positive sentences = 0, negative sentences = 1)	− 24.96	4.18	< .001^***^	− 33.20	− 16.71	.08
SST-wanting (sexual sentences = 0, nonsexual sentences = 1)	− 14.04	3.92	< .001^***^	− 21.77	− 6.31	.03
Scenario task	− 1.96	0.72	.007^**^	− 3.38	− 0.54	.02
Explicit Evaluation Scale of Erotic Stimuli (EEES)	0.47	0.28	.088	− 0.07	1.02	< .01
Sexual distress (FSDS-R)	− 0.21	0.05	< .001^***^	− 0.31	− 0.10	.03
Committed partnership (no = 0, yes = 1)	3.79	.76	< .001^***^	2.30	5.29	.05

*LL* lower limit, *UL* upper limit, *SIDI-F* Sexual Interest and Desire Inventory Female, *STIAT* Single Target Implicit Association Test: D-score, *SST* Scrambled Sentences Test, *FSDS-R* Female Sexual Distress Scale; confidence intervals of the regression coefficients generated with bootstrapping (*N* = 10,000). *N* = 225. *p < .05. ** *p *< .01. ***p < .001

In Step 2, the model explained 52.5% (adjusted *R*^2^, ∆*R*^2^ = .094, *p* < .001) of variance in sexual desire. Sexuality-related personal distress as well as not being in a committed relationship predicted lower sexual desire. The pattern of results concerning the indirect experimental tasks remained unchanged with the exception that the interaction between STIAT-wanting and sexual orientation was no longer significant. Individual effects of the predictors in this model were small (*R*^2^ < .09).

## Discussion

The objective of this study was to investigate the relevance of sexuality-related associations and interpretations for women’s sexual desire. Toward this goal, several indirect measures were used in a sample of women with mixed levels of sexual desire and different sexual orientations. Overall, the study supported the relevance of interpretations for women’s sexual desire while yielding mixed results for the role of automatic associations. The emotion-motivational model (Dewitte, 2016) proposes that automatic appraisals (i.e., associations) are precursors of more conscious, elaborate appraisals (i.e., interpretations) which can trigger sexual desire. This study provided only partial support this model by failing to show substantial correlations between associations and interpretations as well as associations and sexual desire.

### Sexuality-Related Associations and Sexual Desire

To investigate the relevance of associations for sexual desire, we used a STIAT with motivational categories (“I want” vs. “I don’t want”) and four pictures showing heterosexual activities as sexual stimuli. A comparable paradigm has been used in previous studies (e.g., van Lankveld et al., 2018b) and has been shown to differentiate between women with higher and lower sexual functioning. In this study, however, Hypothesis 1 was not supported as more negative associations showed only a trend toward being associated with lower sexual desire in a subsample of women who did not identify as exclusively heterosexual. This finding was unexpected as stimulus material—showing sexual interactions between a man and a woman—was selected to be particularly relevant for heterosexual women. We would like to offer two possible explanations: First, the body of the woman was presented much more prominently in two out of four pictures. Thus, it might be possible that the stimulus material was automatically appraised as sexually relevant only in women who endorsed at least a certain degree of gynephilia (i.e., a sexual attraction toward women). In contrast, exclusively androphilic women (i.e., women who are only attracted toward men) might not have automatically appraised the stimulus as relevant to their sexuality (Snowden & Gray, 2013). A second explanation is that this effect is caused by variables not assessed in this study such as familiarity with sexual stimuli (Træen & Daneback, 2013), hormonal contraception (Bancroft & Graham, 2011), or mood (Dewitte, 2015). As past studies using a STIAT in the study of women’s sexual functioning often excluded women with other than heterosexual orientation (e.g., Borg et al., 2010), more studies are needed to investigate the relevance of this group difference. To address this, researchers might use words (e.g., orgasm, arousal) instead of pictures to prevent characteristics of the stimulus material to bias findings (e.g., Dewitte, 2016). Alternatively, future studies could consider participants’ individual preferences by having them choose their most idiosyncratically relevant pictures from a larger portfolio. To sum up, findings concerning Hypothesis 1 can be interpreted as questioning the contribution of initial, pre-attentive appraisals to self-reported sexual desire in women as such appraisals were not only independent of desire but also not (substantially) related to different measures of sexuality-related interpretations.

### Sexuality-Related Interpretations and Sexual Desire

Three aspects of sexuality-related interpretations were relevant to women’s sexual desire. Regarding Hypothesis 2, results were in line with our expectations in such that lower levels of sexual desire were related to more negative and nonsexual interpretations in the SST. Both variants of SST uniquely added to the explanation of lower sexual desire above other indirect measures as well as interpretations as measured with the FSDS-R. Hence, when comparing our findings with previous studies using self-report questionnaires (e.g., Nobre & Pinto-Gouveia, 2006), these and our results suggest that women with lower levels of sexual desire have a variety of negative interpretations of sexual situations, independent of the measures used (direct vs. indirect). Due to the cross-sectional nature of this study, however, it is possible that this negative interpretation style is a consequence rather than a facilitating factor of lower sexual desire.

Our results provide first evidence for the applicability of the newly developed SST as an indirect measure of negative sexuality-related interpretations in women with varying levels of sexual desire. Compared to women with higher sexual desire, those with lower levels of desire created more sentences that described sex as frustrating, disappointing, or something to be avoided. They also created less sentences that indicated a motivational wanting for sexual cues such as sensuality, intimacy, or passion. As the SST is a short and easy administrable measure, it can be a particularly useful tool in empirical research. If these results can be replicated, future studies might refine and further develop the SST by using sentences that specifically focus on, for example, aspects of sexual functioning (e.g., performance- vs. process-orientation or self- vs. partner-focus).

Regarding Hypothesis 3, and in line with our expectations, individuals with lower sexual desire generated more endings that indicated sexual problems on the scenario task. Our findings correspond to a previous study that included a longer version of the task (Velten et al., 2019) and speak to the validity of the scenario task as an indirect measure of sexuality-related interpretations. While the overall small amount of variance explained by the scenario task can be acknowledged, it significantly added to the explanation of sexual desire over and above other sexuality-related interpretations. While the external rating of scenarios by independent raters is relatively time-consuming, advantages of this tasks are that responses are participant-generated and do reflect idiosyncratic interpretations (Hirsch et al., 2016). This might be especially useful when considering that certain sexual dysfunctions (e.g., genito-pelvic pain) may be associated with a specific set of interpretations. Further, as participants did not receive any information about the dimensions on which their answers were rated, this task may be less affected by demand effects than direct measures.

### Relevance for the Emotion-Motivational Model

With respect to the emotion-motivational model (Dewitte, 2016), these findings suggest that an initial appraisal of a sexual stimulus might not be as relevant for the experience of sexual desire as more conscious, deliberate interpretations. The model acknowledges that the relationship between automatic appraisals and sexual desire is impacted by conscious appraisals as well as attentional processes and perceptions of sexual arousal. Thus, it might be possible that these different pathways do not operate in parallel but in conflicting ways (e.g., negative associations that are followed be more positive interpretations might still lead to sexual desire). For the example provided in the introduction, this may lead to different behavioral outcomes: Even if a woman with high sexual desire does not initially appraise her naked partner taking a shower as something she would “want” sexually, her conscious interpretation of the situation as having the potential for emotional and sexual satisfaction, might still facilitate sexual desire and can lead her to undress and join her partner in the shower. On the other hand, a woman with low sexual desire might consciously interpret the situation as having the risk for frustration and disappointment, and leave the bathroom—despite her initial, pre-attentive appraisal of a sexual “wanting.” The finding that interpretations assessed by different indirect and direct (i.e., sexual distress as measured with the FSDS-R) measures added to the explanation of sexual desire speaks to the multifaceted nature of sexuality-related interpretations and fits into the theoretical framework of the emotion-motivational model in that sexual stimuli need to be interpreted as positive, as more rewarding than nonsexual incentives (e.g., chocolate), and as not indicative of sexual problems, for sexual behavior to occur (Dewitte, 2016).

### Distinction Between Associations and Interpretations

As described in the introduction, cognitive appraisals can be assessed in several ways (e.g., self-report, reaction-time tasks). While a strength of this study is the use of multiple strategies to assess such appraisals, the dichotomy of automatic (i.e., associations) and conscious appraisals (i.e., interpretations) as well as the ability of experimental paradigms (i.e., the STIAT) to provide “pure” information on automatic processes has been called into question (Conrey et al., 2005). For the sake of clarity, we used the term interpretations as a synonym for the strategic process of conscious, deliberate appraisal, although interpretations have also been described as a rule-based process that includes features of both automatic and strategic components (Ouimet et al., 2009). Thus, while we would argue that the scenario task requires at least a certain amount of conscious elaboration (e.g., as answers are provided by entering sentences on a computer keyboard), answers on the SST can be controlled by both automatic and controlled processes: While participants were required to unscramble sentences within 10 s, we did not analyze their reaction times and it might be possible that some participants went with their first, immediate response while others took their time to reflect on their options. We interpreted the paradigms used in this study based on the appraisals most likely to be involved but in reality most tasks are to be impacted by both automatic and controlled processes interacting via feedback loops and not in sequential order (Ouimet et al., 2009).

### Limitations

Although we were able to recruit women of different sexual orientations and ethnicities, the use of convenience sampling led to a sample of women who were (1) more educated and younger than the general population, and (2) showed a somewhat restricted range of sexual desire. Hence, although up to half of the participants scored below a cutoff for low desire, very low desire levels as found in treatment-studies for women with HSDD (e.g., Paterson et al., 2017) were underrepresented. While we were able to conduct comparisons between exclusively heterosexual women and women with other sexual orientations, the lack of exclusively homosexual participants prevented a more elaborate investigation of the role of sexual orientation. The cross-sectional nature of the design prevents any causal interpretation of the obtained results. Relatedly, a potential limitation of our study is that participants completed the experimental paradigms online. Despite the advantages of such an approach (e.g., easy accessibility for participants and the possibility to test a large sample), the lack of an experimenter could potentially have biased our result, e.g., the lack of control over how and when participants completed the tasks. As studies have shown that the assessment of sexuality-related associations with the STIAT depends on context variables (Dewitte, 2015), the remote nature of our study may have affected our findings. However, due to our promising results regarding the measurement of sexuality-related interpretations, the online set-up of our study may not have affected the assessment of more elaborate, conscious appraisals.

### Future Directions

To start with, laboratory-based, cognitive training studies could provide first insights into the causal mechanisms of the processes that both the SST and scenario task targeted, namely negative sexuality-related interpretations. A potential training technique in this context is cognitive bias modification (CBM; Koster et al., 2009; Woud & Becker, 2014). CBM seeks to manipulate pathology-specific, dysfunctional cognitive processes in order to probe their potential causal contribution to symptoms of psychopathology, e.g., by presenting participants a series of ambiguous scenarios which they are asked to complete either consistently in a functional or dysfunctional manner, depending on participants’ training condition (e.g., Mathews & Mackintosh, 2000; Salemink et al., 2009; Woud et al., 2018). Such training techniques have been shown to induce or reduce the trained interpretation bias, to be temporarily stable, unlike, e.g., mood states (e.g., Salemink & Van Den Hout, 2010; Yiend et al., 2005), and to reduce lab induced stress reactions, e.g., after participants have been exposed to stressful film clips (e.g., Woud et al., 2012b) or after a re-activation of a distressing autobiographical event (e.g., Woud et al., 2018; for critical discussions, see Fox et al., 2014 and Grafton et al., 2017). When applying this approach in the present context, future studies could induce a positive, sexuality-related interpretation bias in women with lower sexual desire, and then assess whether changes in interpretation biases generalize, as reflected by more positive scores on the SST and the scenario task post-compared to pre-training. Further, it could be assessed whether such a training is related to increased levels of sexual desire after the training compared to before the training. Relatedly, future studies could further investigate the predictive validity of the SST and scenario task by analyzing changes in women’s negative sexuality-related interpretations pre- to post-cognitive behavioral therapy and analyze, whether the SST and scenario task are sensitive to detect potentially changed interpretations during therapy.

### Conclusion

This study underscores the multifaceted nature of sexuality-related appraisals and provides partial support for the theoretical framework of the emotion-motivational model (Dewitte, 2016). Interpreting sexual cues as positive, as more rewarding than nonsexual incentives, and as not being indicative of sexual problems was related to higher sexual desire in a sample of women with different sexual orientations. In contrast, the predictive value of pre-attentive automatic associations for sexual desire was not supported. Our results support the use of indirect measures to assess sexuality-related interpretations. Future research should assess the potentially causal role of sexuality-related interpretations in women with sexual low sexual desire.

## Electronic supplementary material

Below is the link to the electronic supplementary material.

Supplementary material 1 (XLSX 13 kb)

## Data Availability

The dataset used for the analysis can be accessed at the following website: osf.io/ruksm/.

## Footnotes

1 The original German stimuli were “Das will ich” and “Das will ich nicht” as attribute category, the target category was “Sex,” the positive words were Humor, Gesundheit, Geschenk, and Frieden and the negative words were Hass, Krankheit, Schmerz, and Krieg, respectively.

2 A second D-score was calculated marking all responses < 300 ms and > 3000 ms as missing. Both D-scores were highly correlated (*r* = .99, *p* < .001) and the pattern of results was identical; thus, only the results from D-scores via Bluemke and Friese (2008) are reported.
